# Comparisons of Efficacy between Autograft and Allograft on Defect Repair *In Vivo* in Normal and Osteoporotic Rats

**DOI:** 10.1155/2020/9358989

**Published:** 2020-03-03

**Authors:** Chris H. Dreyer, Marina Rasmussen, Rasmus Hestehave Pedersen, Søren Overgaard, Ming Ding

**Affiliations:** Orthopaedic Research Laboratory, Department of Orthopaedics and Traumatology, Odense University Hospital, Institute of Clinical Research, University of Southern Denmark, 5000 Odense C, Denmark

## Abstract

*Introduction*. In the field of orthopaedic surgery, the use of osteogenic material in larger defects is essential. Autograft and allograft are both known methods, and autograft is believed to be the best choice. But autograft is associated with additional invasive procedures which can prove difficult in fragile patients and can cause local side effect after bone harvest. For feasible purposes, the use of allograft is hereby rising and comparing efficacies, and the differences between autograft and allograft are essential for the clinical outcome for the patients.

**Method:**

24 female Norwegian brown rats were included, 12 normal rats and 12 induced with osteoporosis (OP). OP inducement was verified in vivo by bone volume fraction (BV/TV) at 90 days after ovariectomy (OVX). The primary surgery in each rat consisted of a 2.5 × 3 mm hole in the proximal tibia, bilaterally. Autograft and allograft were randomly allocated in the right and left tibia. After an observation of 21 days, the rats were sacrificed. Tibia samples were harvested, micro-CT scanned for bone inducement and microarchitectural properties, and then embedded for histology.

**Results:**

The OP induction was verified three months after the OVX by a reduction of 68.5% in the trabecular bone BV/TV compared to normal bone. Microarchitectural analysis and histology showed no significant differences in the bone-forming capabilities between autograft and allograft in normal or osteoporotic bone after 3 weeks.

**Conclusion:**

This study did not demonstrate any difference between autograft and allograft in a normal or osteoporotic rat tibial defect model after 21 days, suggesting allograft is a good alternative to autograft.

## 1. Introduction

Bone loss and defects can be caused by trauma, infections, or following arthroplasties. They are categorized as one of the biggest clinical challenges in orthopaedic surgery [1]. Implant surgery is the general term and includes a large portion of intervention surgeries like the repair of bone defects in orthopaedic surgery, neurosurgery, oral, and maxillofacial specialties. These surgeries are estimated to be performed over 2 million times a year and hereby have a high impact on the patients and the economic outcome [2].

Autograft bone is considered as a “living” material bearing osteogenic, osteoinductive, and osteoconductive properties [3] and should provide the best treatment, where allograft consists of inactive “dead” bone with mainly osteoconductive properties [4]. However, harvesting autograft bone is an additional invasive procedure, and the amount available is often insufficient. Autograft collected from the iliac crest bone is termed autogenous iliac crest bone graft (AICBG) [3]. This procedure can be associated with morbidities, such as blood loss, donor site pain, risk of infections, and nerve injuries. Failure rates in autograft bone graft surgeries have been shown to be 50% caused by different types of harvesting, handling, implantation method used, and differences between patient conditions and bone vitality [5]. Due to these complications and high costs, allogenous bone material is often used as an alternative graft material. Allogenous bone is gathered conveniently without side effect from other patients. But this graft material has mainly osteoconductive effect [6] and has the potential risks of disease transmission, bacterial infections, autoimmune host response, and graft host nonunions. These side effects are more severe but are however extremely rare [7]. Theoretically, the gentlest treatment is the allograft. With increasing elderly and fragile patients, it is essential to acquire knowledge by comparing them in a relevant defect model, both in regular and fragile bone structures. An alternative to diminish the need for harvesting live bone graft is substitute [3, 8]. The approach has been wide from the use of stem cells from different tissues [9, 10] to combinations with growth factors in animal and clinical models [10, 11]. Yet, no substitute has shown a stable effect to replace all the procedures using autograft and allograft in the clinic.

Osteoporosis is an age-related rising disease and is a major public health problem related to increasing age in the population and hereby an increasing prevalence and treatment cost globally of 20-30% in 2030 [12]. Osteoporosis is a bone disease with pronounced reduction of bone mineral density. This is caused by the osteoclasts (OC) that resorb more bone than the osteoblast (OB) can produce, disturbing the stability in the bone remodelling unit (BMU), which leads to an imbalance between the bone resorption and formation and eventually bone loss [13]. This states the importance of using verified models and testing both normal and fragile bone structures for the best clinical correlation to enhance the correlation to the relevant situation.

Thus far, there is limited knowledge on the differences between autograft and allograft in bone defect repair.

This study is aimed at investigating the effects of autograft and allograft on tibial defect repair in vivo in both normal and osteoporotic rats. Particularly, the in vivo longitudinal microarchitectural changes postoperatively, at 7 days, 14 days, and 21 days. The evaluation was performed with micro-CT scans at all time points and histomorphometric evaluation after euthanization at 21 days. The primary objective was to evaluate the bone formation between autograft and allograft. The secondary objective was to verify the induction of osteoporosis 90 days after the removal of the ovaries. It was hypothesized that there were no differences in the defect repairs between autograft and allograft in both the normal and the osteoporotic rats, which can help reduce the use of autograft clinically in defect models in both normal and fragile bone structures and decrease the need for additional invasive harvesting procedures.

## 2. Materials and Methods

### 2.1. Animal Model

Twenty-four female brown Norwegian inbred (BN/SsNOlaHsd) rats were included in the study. Both the normal and osteoporotic rats were 4 months of age with an average weight of 190 ± 25 g. They were housed and acclimatized 2 months prior to surgery at the Biomedical Laboratory, University of Southern Denmark. The controlled environment had a temperature of 21-28°C, humidity of 40–60%, and lights on between 6 a.m. and 6 p.m, with access to sterile water and normal or Ca-deficient diet ad libitum, respectively. The cages had sawdust flooring and bedding material. The animals were observed every day for changes in behavior or signs of discomfort by either the animal technicians or the researcher.

### 2.2. Animal Approval

All the experimental procedures were performed in accordance with the Danish Animal Research guidelines. This experimental protocol was approved by the Danish Animal Experiments and Inspectorates (no. 2011/561-1959). This article follows the Animal Research: Reporting of In Vivo Experiments (ARRIVE) guidelines.

### 2.3. Bone Graft Materials

#### 2.3.1. Autograft

The autograft bone material was harvested from bilateral rat tibiae during the drilling process of the tibia defect surgery. The bone was treated sterile, and the chips were of approximately 0.5-1 mm. In the filling of the tibia defect, the normal bone had sufficient volume from the drilling procedure to fill the gap. However, for the osteoporotic rats, the defect needed additional filling by bone material from the tail vertebrae. For the osteoporotic rats, additional bone material was acquired from the tail vertebrae. With a small incision 2 mm distal from the attachment of the tail to the body, soft tissue was removed by surgical equipment and sectioned into chips. The need for additional bone was due to the pronounced bone loss of bone mineral density after osteoporotic inducement. This method is well known for the harvest of autologous bone use in the rat model [14].

#### 2.3.2. Allograft

This was produced from one healthy brown Norwegian female rat. It was euthanized with pentobarbital according to the guidelines, and the condyles of femur, tibia, and humerus were harvested under sterile conditions. Additional soft tissue was carefully removed. The bone was prepared by bone mill (Ossano Scandinavia ApS, Stockholm, Sweden). Trabecular bone structure was divided into chips with a diameter of 0.5-1 mm and stored at 80°C. Before use, the frozen bone was thawed for 30 minutes and added by surgical standards to the defect. All these procedures were performed under an aseptic condition in the small animal surgery room in the Biomedical Laboratory, University of Southern Denmark.

### 2.4. Study Design

**Figure 1:**
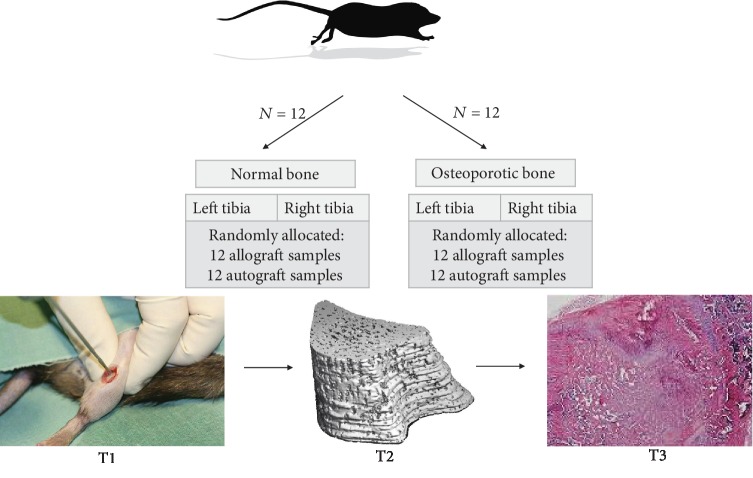
Illustration of study design. 24 rats were included and divided into normal or osteoporotic groups. 12 rats each. Each rat had a group of autograft and allograft in the left and right tibia for a total of 24 samples from normal bone and 24 samples for the osteoporotic bone. T1 (week 0): day for surgery; T2 (weeks 0, 1, 2, 3): micro-CT scans; T3: histology section.

A paired longitudinal study design was used, with one control group and one intervention group in each tibial bone. A total of 24 Norwegian inbred (BN/SsNOlaHsd) female rats were divided into two groups of 12 each in the normal and osteoporotic bones. Autograft and allograft were blinded and randomly allocated in the right and left tibial defect and furthermore blinded in the evaluation stage (Figure 1). At 13 weeks prior to tibial surgery, 12 rats received an ovariectomy (OVX) for the osteoporosis induction. The allograft material was thawed 30 minutes before usage, and autograft was prepared and collected under the anesthesia for the primary surgery of the proximal tibia defect. At day 0, day 7, day 14, and day 21 postoperatively, micro-CT scans were performed for ongoing evaluation. At sacrifice, bilateral proximal tibial defect samples were harvested for histology and histomorphometry.

### 2.5. Surgical Procedures

#### 2.5.1. Ovariectomy (OVX)

12 rats were anesthetized with 0.3 ml/100 g body weight hypnorm (VetaPharma Ltd., Leeds, UK) and dormicum (B. Braun, Melsungen, Germany) mixture, subcutaneously (s.c.) until sedation. Before surgery, the rats were given buprenorphine (Temgesic, RB Pharmaceuticals Limited, Berkshire, UK) 0.2 ml/100 g body weight s.c. for pain-relieving maintenance.

The back of the rat was shaved and disinfected with iodine and ethanol (70%). A sharp incision of approximately 1 cm was made over the caudal part of the back and bluntly dissected until the fascia. By penetration of the cavity, the ovary was harvested with a tweezer, and ligation was made beside the ovary with 5.0 ethilon suture and removed. The wound was closed in two layers. All operations were performed at the same time of the day and on the same location. Postoperatively, the rats were analgesized with buprenorphine (Temgesic, RB Pharmaceuticals Limited, Berkshire, UK), 0.2 ml/100 g body weight s.c. in intervals of 8 hours for the following 3 days. After ovariectomy, the rats were given a special diet with low calcium and water ad libitum. The inducement of the osteoporotic bone structure was verified after 12 weeks, and primary tibial defect surgery could be initiated.

#### 2.5.2. Tibia Proximal Defect

Anesthetic protocol during surgery followed the methodology of the OVX surgery.

Bilateral proximal tibial defects were made in all rats using the standard surgical procedure. Both limbs were isolated, shaved, and disinfected with iodine vet (Kruuse Vet, Denmark) and 70% ethanol. Sharp incision and blunt exploration presented the medial side of the proximal tibia, where a cylindrical defect of 2.8 mm and depth of 3 mm till opposite cortical shell were created based on the results from a pilot study. The surgery for allograft group was performed firstly, due to the collection of autografts from both legs. In total, 48 cylindrical defects were created and filled with either autograft or allograft scheduled by randomization. The wound was closed in two layers with suture 4.0. Postoperatively, the rats were analgesized with buprenorphine (Temgesic, RB Pharmaceuticals Limited, Berkshire, UK), 0.2 ml/100 g body weight s.c. in intervals of 8 hours for 4 days.

### 2.6. Micro-CT Scanning and Microarchitectural Analysis

The bilateral proximal tibial regions were scanned in vivo with a high-resolution microtomographic system (vivaCT 40, Scanco Medical AG, Brüttisellen, Switzerland).

In vivo micro-CT scanning for evaluation of bone growth development in normal and osteoporotic rats was performed at the following 4 time points: the day prior to surgery (day 0) and then at 7, 12, and 21 days postoperatively.

Furthermore, to verify the osteoporosis induction model, a longitudinal evaluation was conducted at 3 time points: (1) before OVX surgery to establish a baseline of bone mineral density (day 90), (2) before the tibia surgery (day 7), and (3) after tibial surgery (day 0).

During in vivo scanning, the rats were anesthetized by isoflurane in a closed box system, 1 L/min oxygen and 4 ml/min isoflurane (IsoFlo vet, Abbott Laboratories Ltd, Berkshire, England) for 6 minutes, with regulation according to the reflexes of the rat. After full sedation, the rats were placed in an animal holding bed covered with a mask with a running supply of oxygen and isoflurane according to protocol. The area of interest was fixated for accurate scans. The images were scanned in a high resolution resulting in a 3D reconstruction voxel sizes of 10.5 × 10.5 × 10.5 *μ*m^3^ (2048 × 2048 × 2048 pixels) for 500 slices for representative evaluation of each defect area. Scanning time for each specimen was 30 minutes.

The parameters specified by this scan included microarchitectural properties of the trabecular bone for the confirmation of osteoporosis induction and bone enhancing effect of the grafts [15, 16]. This included bone volume/tissue volume (BV/TV), structure model index, connectivity density (CD), trabecular thickness (TbTh), trabecular separation (Tb.Sp), degree of anisotropy (DA), bone surface density, bone surface-to-volume ratio (BS/TV), apparent density, and material density.

### 2.7. Histology and Histomorphometry

21 days after surgery, the rats were scanned and sacrificed with overdose pentobarbital according to the animal license protocol. Bilateral proximal tibiae including graft material and bone were fixated in formaldehyde (4%) and changed to phosphate-buffered saline (PBS). After the dehydration and decalcification, the samples were fixated and paraffin-embedded. The samples were sectioned in 3 consecutive slices with a thickness of 3-4 *μ*m and separation of 500 *μ*m. All 3 sections were stained with hematoxylin and eosin (H&E).

**Figure 2:**
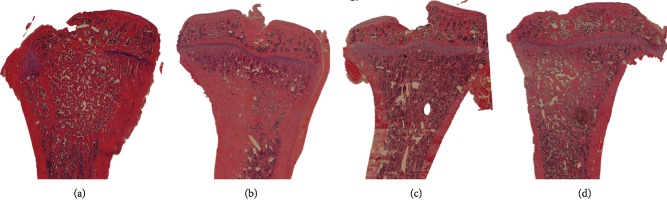
Sections and illustration of the defect placement in the tibial bone. (a) Autograft in normal bone. (b) Allograft in normal bone. (c) Autograft in osteoporotic bone. (d) Allograft in osteoporotic bone.

The region of interest (ROI) for histomorphometry was characterized as the original tibial defect area compared between autograft and allograft (Figure 2). Within the predefined ROI, the volume fractions were calculated by Cavalieri's principle using verified stereological software (newCast Visiopharm, Denmark) for point counting, with 300-600 hits per section with Olympus BX 51 Microscope (Ballerup, Denmark) [17, 18].

The tissue within the ROIs of the HE-stained sections was classified as bone, fibrous tissue, miscellaneous, muscle, or marrow. The bone volume was calculated as the amount of bone hits divided with the total hits and stated in percent.

### 2.8. Statistical Analysis

Two samples two-tailed *t*-test and one-way ANOVA were used to compare possible differences between groups with GraphPad Prism v. 7 (GraphPad Software, Inc.). A *p* value of less than 5% was considered significant.

The sample size included at least 10 defects for each graft. We chose to include 12 rats in each group due to the risk of dropouts. The calculation error of the first kind was set to 1.96/95% and error of the second kind to 0.84 due to the selected power of 80%. Minimal relevant difference and standard deviation were both set to 70% [19].

## 3. Results

### 3.1. Animal Observation

In total, four of the 24 rats died during the 3 postoperative weeks, consisting of 2 from each group. Three of them died as a response to the anesthesia during in vivo micro-CT scanning, and one died caused by an infection. The remaining rats were included into the study. During the observational period of the experiment, the animals were observed daily by an animal technician or the researcher for any signs of discomfort or violation of the animal license. No significant weight change was observed in either group after tibia defect surgery. The rats exposed to OVX gained weight during the first 12 weeks from 193 ± 8 g to 224 ± 44 g (*p* < 0.001).

### 3.2. Three-Dimensional Microarchitectural Properties

#### 3.2.1. Induction of Osteoporosis in Rats

After 12 weeks, OVX-treated rats had a decrease in bone volume fraction, connectivity density, bone surface density, and apparent density (*p* < 0.001) compared to normal bone. Structure model index increased from typical plate, -1.4, to typical rod, 3.3. Trabecular separation, degree of anisotropy, material density, and bone surface-to-volume ratio increased (*p* < 0.001). Trabecular thickness did not have any significant change (*p* < 0.097) (Table 1).

#### 3.2.2. Microarchitectural Changes with Autograft and Allograft Treatments

**Figure 3:**
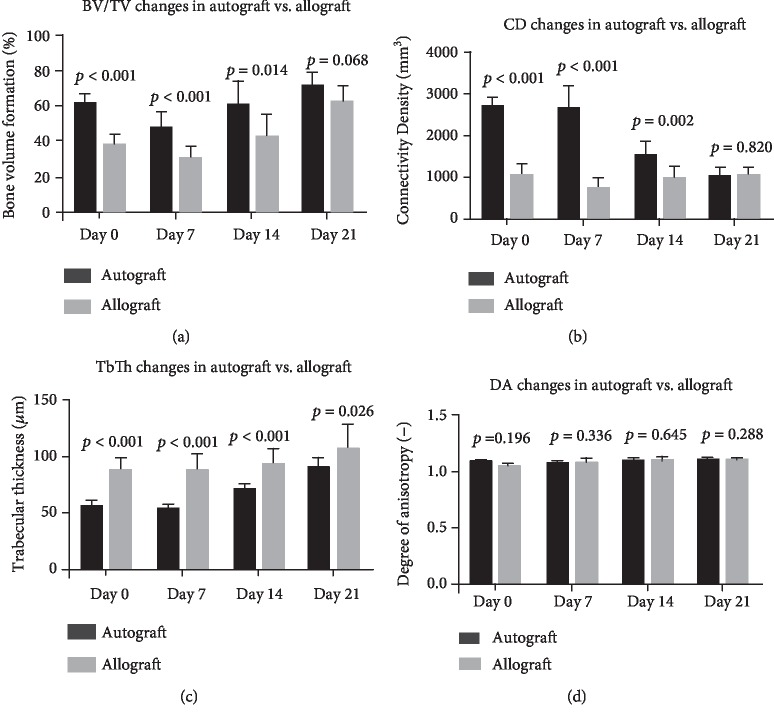
Microarchitecture properties of defect treated with autograft vs. allograft in normal bone. BV/TV: bone volume/tissue volume; CD: connectivity density; TbTh: trabecular thickness; DA: anisotropy. *p* < 0.05 is considered significant.

**Figure 4:**
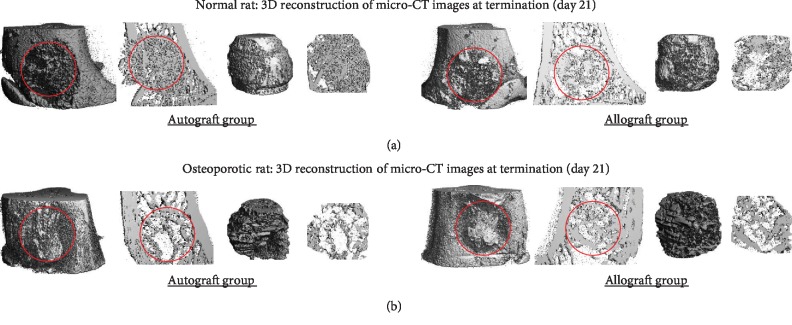
Representative 3D reconstructions of micro-CT images at termination (day 21) for both autograft and allograft groups and in normal (a) and osteoporotic (b) rats are illustrated from the same tissue as Figure 2. Red circle indicates where original defect holes were created. At the right side of each image is the newly generated bone mass within the hole. All images are displayed as whole mass and thin layer of 10 slices (105 micrometers).

*(1) Changes in Microarchitecture in Normal Bone*. BV/TV was decreased in allograft defects compared to autograft on days 0, 7, and 14 (*p* < 0.05). However, on day 21, there was no statistical difference between the two groups (Figure 3). Representative 3D reconstructions of micro-CT images have been displayed in Figure 4.

The same trend applied to the connectivity tissue with decreased value on days 0, 7, and 14 (*p* < 0.05) but no difference on day 21. Trabecular thickness was increased at all time points in the allograft groups (*p* < 0.05) and no difference in the degree of anisotropy (Figure 3).

**Figure 5:**
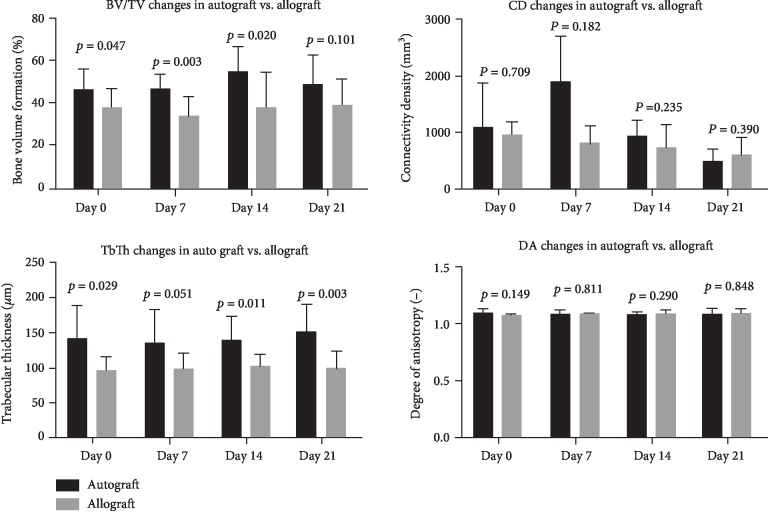
Microarchitecture properties of defect treated with autograft vs. allograft in osteoporotic bone. BV/TV: bone volume/tissue volume; CD: connectivity density; TbTh: trabecular thickness; DA: anisotropy. *p* < 0.05 is considered significant.

*(2) Changes in Microarchitecture in Osteoporotic Bone*. BV/TV of osteoporotic bone displayed decreased bone in the allograft group on days 0, 7, and 14 (*p* < 0.05) and no difference after 21 days (Figures 4 and 5).

Trabecular thickness was significantly higher in the autograft group on days 0, 14, and 21 (*p* < 0.05) but no difference on day 7. Connectivity density and degree of anisotropy had no significant difference at any time point (Figure 5).

### 3.3. Histology and Histomorphometry

#### 3.3.1. Histology

New bone formation was observed in the defect area in all samples. It was not possible to distinguish the difference between remnants of graft and new bone, whereas the total bone volume within the defect area was calculated as bone within the ROI (Figure 1, T3).

#### 3.3.2. Histomorphometry

Bone volume showed no significant difference between the autograft and allograft groups within the normal and osteoporotic bone. When comparing autograft and allograft from the normal bone with autograft and allograft in the osteoporotic bone, there was significantly decreased amount of bone at 21 days of evaluation (*p* > 0.001).

**Figure 6:**
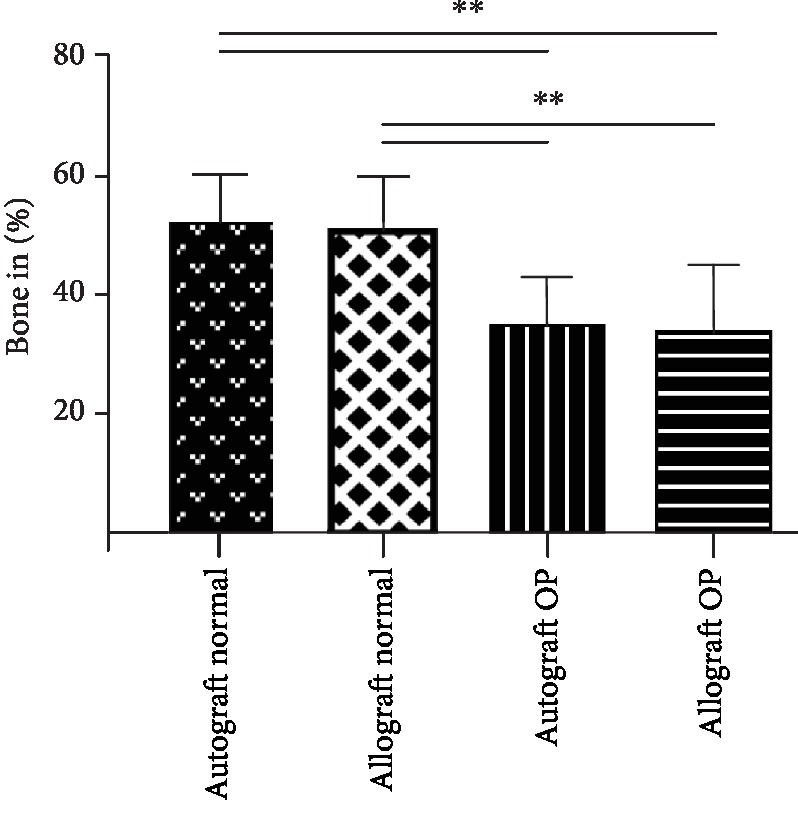
Histomorphometric evaluation of bone formation in normal and osteoporotic bones: ^∗∗^*p* < 0.001.

The mean formation of new bone including graft material in the defect in normal bone was 53% for autograft, whereas 51% for allograft. In osteoporotic bone, allograft had the highest bone volume with a mean of 35%, whereas autograft had 33% (Figure 6). There was no significant difference between any other measured parameters within the defect (*p* > 0.05).

## 4. Discussion

This study compared the effects of autograft and allograft in a tibial defect rat model for the best defect repair in normal and osteoporotic bones. The hypothesis was that there would be no difference in bone formation using either autograft or allograft, whether it is used in normal or osteoporotic bone. The results from the micro-CT or histomorphometry showed no significant difference in the use of autograft and allograft in a tibial bone defect after an observation of 21 days, in neither normal nor osteoporotic bone structures. However, micro-CT scans showed a decreased amount of new bone in the allograft group at 0, 7, and 14 days.

When studies compare their inventions, there is some disagreement whether autograft or allograft should serve as the golden standard. The difference is primarily between the academical and clinical opinions and which kind of defect is used in the study [20, 21]. By making a comparison within the same model, a valuable information can be obtained for the academic purpose and for clinical application, especially, due to the increasing focus on substitute materials to overcome the challenges in using graft materials and their limitations [22].

When evaluating the results from this study, it should be noted that in both normal and osteoporotic bones, the BV/TV on the micro-CT scan is significantly lower in the allograft group on day 0, which could be due to the process of filling the defect. Allograft was obtained from normal bone and the autograft from the same animal with either normal or osteoporotic bone. The trabecular thickness is lower in the autograft group in the normal bone but higher in the osteoporotic bone. In theory, this should have been with opposite results as for the decreased density in the osteoporotic bone. However, it could be from the effect of the bone mill and the size and mobility of the chips. The interesting aspect is that the development from day 0 to day 21 is faster in the allograft group with 65.8% increase vs. only 16.4% increase in the autograft group. At day 21, there is no difference between autograft and allograft quantified by histomorphometry or microarchitectural analysis.

Histomorphometry revealed on day 21 a decreased general bone formation when trying to use graft materials in osteoporotic bone (Figure 6). Again, it should be noted that the allograft is from a healthy donor, and yet it provides significantly lower regeneration than it does in normal bone. This perfectly emphasizes the need to test graft materials or medical devices in the osteoporotic bone to be able to evaluate their full efficacies. However, this test of graft material in both normal and osteoporotic bone tissues seems to be lacking in regenerative potential.

Induction of osteoporosis in a rat model has previously been verified, with an illustration of the importance of observation until induction. Kinney et al. [23] investigated the changes in the trabecular bone due to OVX and found that the OVX procedure leads to an immediate and continuous decrease in trabecular bone, and after 50 days, the OVX rats had lost 50% of their bone volume with no rebound effect. Campbell et al. [24] established a detailed longitudinal time course of bone loss in OVX rat model in twelve weeks. The results of this study indicate that microarchitectural changes occur within the first 12 weeks after OVX in the rat model. Hereby, with the observation of 21 days, there are no expectations of a rebound effect.

The current knowledge in the use of allograft and autograft is that studies verify different outcomes dependent on location. In the cranioplasties, allograft has been shown to be superior [25]; in anterior crucial ligament (ACL), autograft showed better effect on bone formation [26], whereas in the posterior crucial ligament (PCL), the results are equal between the 2 grafts [27, 28]. Hence, when comparing results for clinical implementation, using the correct graft is essential for the correct comparisons. Optimal results will be obtained by using both grafts for both positive and negative controls. This will make the clinical impact higher and will provide overall convincing results.

When facing a clinical implementation, it is furthermore required to focus on an economic and patient-related outcome. The cost of using autograft is reported to be lower in, for example, ACL surgeries [26], but the possible side effect in harvesting autograft is associated with rather severe side effects [3]. This gives the dilemma for choosing methodology when harvesting autograft, but it even further requests the need for another substitute to replace both graft materials. Current tissue engineering and biomaterials with stem cells might provide new hope to bone regeneration.

The strengths of this study are the standardized tibia defect model in rats and the well-verified induction of osteoporosis. Yet, nothing in this paper relies on previous results, and the osteoporosis induction is verified by the same scanner that evaluates the results in the intervention groups. This means that the reliability of the results of this study increases. Furthermore, results and same methods are tested in different types of the bone structure allowing for the best comparison of graft material, especially as the allograft in normal and osteoporotic bone is from the same donor.

Limitations are the lack of an empty defect that could demonstrate the baseline effectiveness of the bone regenerative potential without any bone grafts in both normal and osteoporotic bone. However, the focus of this study is to compare potential efficacies between the graft materials and not according to a specific baseline for the design model. Another limitation is the significant difference on day 0 between the groups. But when the evaluation is limited to 21 days and the allograft manages to provide the same results in such a short duration, and hence, the results are validated in this model.

## 5. Conclusion

This study concludes that autograft and allograft have similar bone-forming capabilities with an observation of 21 days in a rat tibial defect in a rat model, suggesting allograft could be a good alternative to allograft. Furthermore, OVX surgery for lasting osteoporosis induction in the rat model is a feasible method.

## Figures and Tables

**Table 1:** Assessments of changes in microarchitectural properties with osteoporosis induction after OVX performed on day 90. Day 0 is the day of the tibial surgery.

	Bone volume fraction (%)	Structure model index (-)	Connectivity density (mm^−3^)	Trabecular thickness (*μ*m)	Trabecular separation (*μ*m)	Degree of anisotropy (-)	Bone surface density (mm^−3^)	Bone surface-to-volume ratio (mm^−3^)	Apparent density (mg/cm^3^)	Material density (mg/cm^3^)
Day 90	54 ± 8	-1.4 ± 1.6	1324 ± 282	63 ± 5	56 ± 9	1.4 ± 0.03	19.1 ± 1.4	34 ± 5	505 ± 52	863 ± 16
Day 7	19 ± 12	3.3 ± 0.9	248 ± 230	62 ± 3	160 ± 50	1.4 ± 0.06	7.8 ± 4.3	44 ± 4	234 ± 100	888 ± 9
Day 0	17 ± 12	3.3 ± 0.9	210 ± 198	59 ± 3	173 ± 55	1.4 ± 0.58	6.8 ± 4.6	46 ± 4	217 ± 105	884 ± 12
ANOVA	*p* < 0.001	*p* < 0.001	*p* < 0.001	*p* = 0.097	*p* < 0.001	*p* < 0.001	*p* < 0.001	*p* < 0.001	*p* < 0.001	*p* < 0.001

## Data Availability

The data for this study were analyzed by histomorphometry and all data are stored at the Orthopaedic Research Laboratory, Department of Orthopaedics & Traumatology, Odense University Hospital, Institute of Clinical Research, University of Southern Denmark in datafiles from VisioPharm, Denmark verifying every count and statistics made for the analyses incorporated in this study. Micro-CT scans are large files stored at TB tapes. All data used to support the findings of this study are available from the corresponding author upon request.
